# Nutritional needs in the professional practice of swimming: a review

**DOI:** 10.20463/jenb.2017.0030

**Published:** 2017-12-31

**Authors:** Raúl Domínguez, Antonio Jesús-Sánchez-Oliver, Eduardo Cuenca, Pablo Jodra, Sandro Fernandes da Silva, Fernando Mata-Ordóñez

**Affiliations:** 1. Department of Sciences of the physical activity and Sport. Faculty of Sciences of the Public. Alfonso X el Sabio University. Madrid España; 2. Department of Sciences of the Sport. Faculty of Sciences of the Sport. Pablo Olavide University. Sevilla España; 3. Department of Physical Education and Sport. Faculty of Sciences of Education. University of Seville. Sevilla España; 4. Department of Sciences of the physical activity and Sport. Faculty of Sciences for Health. Pompeu Fabra University. Mataró. Barcelona; 5. Physical Education Department. University of Lavras. Lavras Brazil; 6. NutriScience. Córdoba España

**Keywords:** Ergogenic aids, Exercise, Nutrition, Aquatic Sport, Supplement, Swimmers

## Abstract

**[Purpose]:**

Swimming requires developing a high aerobic and anaerobic capacity for strength and technical efficiency. The purpose of this study was to establish the nutritional requirements and dietary strategies that can optimize swimming performance.

**[Methods]:**

Several related studies retrieved from the databases, Dialnet, Elsevier, Medline, Pubmed, and Web of Science, through keyword search strategies were reviewed.

**[Results]:**

The recommended carbohydrate intake ranges between 6-10-12 g/kg/d, protein 2 g/kg/d, and fat should surpass 20-25% of the daily intake.

**[Conclusion]:**

Performance can be optimized with a hydration plan, as well as adequate periodization of supplements, such as caffeine, creatine, sodium bicarbonate, B-alanine, beetroot juice, Vitamin D, bovine colostrum, and HMB.

## INTRODUCTION

Aquatic sports include a variety of sport disciplines with varying degrees of metabolic, strength, and technical demands, while together ensuring that body displacement takes place in water. The aquatic sport that awards the most Olympic medals is swimming. There are four different styles: freestyle, butterfly, backstroke, and breaststroke, all with different competitive distances (50, 100, 200 meters). In addition, the medley is a modality where all four different styles are used, competing in distances of 200 and 400 meters. The relay includes 4 x100, 4 x 200 freestyle, and 4 x 100 medley, as well as competitions of 400, 800, and 1500 meters ^1^. 

Due to the difficulty of displacement in water, it is important to apply techniques that make use of propulsive forces and reduce drag forces produced by the water. The significance of technique over performance in swimming, justifies in part, the high volume of training that swimmers need to endure in the swimming pool. Techniques can influence the swimmer’s fatigue, as well as diminish the efficiency of propulsive force applications ^2^. Possessing and maintaining good technique in situations of fatigue is key to success in swimming. 

As the displacement speed of the swimmer increases, the drag forces also intensify, requiring an increase in strength as performance levels rise. Therefore, swimmers incorporate strength training in addition to their swimming program in the pool ^3^. 

At the metabolic level, all three energy systems (the high energy phosphagen system, anaerobic glycolysis, and aerobic metabolism) are involved in all the different swimming competition disciplines, which range between 20 seconds (50 m), and 15.5-16 minutes (1500 m) ^4^. The contribution of the different energy systems is dependent on the distance of the competition. While the 200-meter modality utilizes the aerobic energy system at 65% energy expenditure ^5^, the 400-m modality can utilize around 81% of energy expenditure ^6^. In contrast, during shorter distance races, (50 and 100 m), the aerobic system only contributes 15.3% and 33.3% of energy expenditure respectively ^7^. 

With the goal of maximizing the high energy phosphagen system in swimming, it is necessary to develop a specific high-intensity training program ^8^, as well as one focused on improving maximal oxygen consumption (VO2max). In addition to interval training sessions to help improve the high energy phosphagen metabolism, the anaerobic glycolysis, and the VO2max, the swimmers must perform continued long duration with low-intensity training, in order to improve both the efficiency and efficacy of their swimming technique ^1^. 

During longer distance competitions ^9^ and interval training sessions ^10^, the glycogen stores in the muscles can be depleted, possibly causing a decrease in performance, and consequently lowering training adaptation. Furthermore, there may be a resulting increase in proteolysis ^11^, compromising nitrogen balance, propulsive strength capacities, and the super compensation processes. 

It has been suggested that by identifying the limiting factors of performance in a sport modality, like swimming, nutritional strategies and goals can be developed, to help establish a nutritional intervention plan for the sport ^12^. An adequate nutritional and supplementation plan, that takes into account the characteristic of the sport modality, can help improve the health and performance of the athletes ^13^. The aim of this review is to establish nutritional objectives and strategies to help optimize swimming performance by identifying factors that limit performance in swimming. 

## METHODS

The present literature review has been performed after thorough search of the following databases: Dialnet, Elsevier, Medline, Pubmed, and Web of Science through keyword search strategies. The terms included in the browsers can all be found in the Thesaurus Medical Subject Headings (MeDH) developed by the U.S. National Library of Medicine. The terms were: swim, swimmer, swimming, and aquatic sport in combination with supplement, nutrition, sport nutrition, and ergogenic aids. 

## RESULTS and DISCUSSION

### Energy requirements and macronutrients

One of the main focuses in swimming training is high volume training ^3^ that includes 1-3 daily sessions. Training sessions are performed both in the pool and on land with strength training, core, or cross training ^14^. 

Occasionally, the high training volume results in below adequate energy intake for the swimmer to maintain adequate energy balance, with intake that can reach the 2400 kcal/day on extreme training periods ^15^. It is worth mentioning here, that energy deficits maintained for long periods of time, could result in decreased basal metabolic rate, cause alterations in hormonal functions, disrupt menstrual cycles in women, as well as increase the risk of injury and disease ^16^^,^^17^. It has recently been found that high level female swimmers who experience menstrual dysfunctions show a decrease in thyroid hormones and growth factors, coupled with a decrease in speed in the 400-m test after a 12-week training period ^18^. 

Regarding carbohydrate intake, it is necessary to point out that a 10% reduction in daily carbohydrate intake has been shown to lower performance in a 365-m freestyle test in swimmers, while an increase of 10% led to improvements in both tests of 91.5 and 365 m ^19^. Although these results may suggest a need to follow a diet rich in carbohydrates, it is worth highlighting another study that did not observe any differences in performance among a group of swimmers who followed a diet of 6 g/kg/day compared to a 12 g/kg/day carbohydrate diet intake, after following a moderate training volume ^20^. Carbohydrate requirements should be periodized according to training demands ^14^. Beginning a training session with low carbohydrate availability (due to low carbohydrate intake since the last training session), causes a higher metabolic stress that can optimize training adaptations ^3^^,^^16^, as long as the training sessions are of moderate intensity. 

Therefore, for athletes who follow a moderate- to high-intensity training program, carbohydrate intake should be kept in ranges of 6-8 g/kg/day on days when training sessions are of high volume and low intensity, or high intensity and very low volume. On days of moderate or high volume with high intensity, carbohydrate intake should be 10-12 g/kg/day ^21^. It is important to point out that when engaging in high intensity sessions with the recommended amount of carbohydrate intake, it will keep the immune system in a better state through an increase in the salivary immunoglobulin response ^22^, decreasing the risk of respiratory tract infection ^23^. However, these general recommendations should be fine-tuned with individual consideration of total energy needs, specific training needs, and feedback from training performance

Recommendations for carbohydrate timing are the same as in other sport modalities, except before high-intensity sessions, where training sessions require high CHO-availability ^3^. Following the recommendations of other sports modalities, it could be recommended that 200- 300 g ^24^ or 1-2 g/kg be consumed 3-4 hours prior to the training sessions ^25^. Carbohydrates, especially high on the glycemic index must not be ingested 45 minutes prior to exertion, to avoid reactive hypoglycemia ^26^. 

Carbohydrate intake during exercise favors glycogen oxidation in the muscle, keeping blood sugar levels stable, while preserving glycogen stores during longer periods of time, and avoiding protein catabolism ^27^. Therefore, carbohydrate intake during high-intensity training sessions should be favored with intakes up to 90 g/hour ^28^ in a 2:1 ratio of glucose and fructose ^29^. 

Studies have shown that open water competitions have the capacity to deplete muscle glycogen storages ^9^. Because intake of carbohydrates during competition is difficult, athletes are recommended to eat a super-compensation carbohydrate diet ^10^. Athletes should consume 10-12 g/kg/day of carbohydrates 48 hours prior to a competition, the same amounts as recommended prior to high-intensity interval sessions ^30^. Then, 4 hours before a competition the intake should be at 1-4 g/kg/ day. 

Protein requirements for the athletes range between 1.2-2 g/kg/day ^31^. Due to the strength requirements in swimming, the recommended intakes should be on the higher limit that is 2/g/kg/day. The dietary protein intake, which only provides a very small amount to the energy metabolism, especially in situations where glycogen stores are depleted ^3^^,^^16^, is necessary for the synthesis of new myofibrillar tissue, as a response to strength training, as well as sarcoplasmic, and mitochondrial proteins in response to endurance, and interval training ^32^. 

It must be emphasized, that the protein food must be of high bioavailable value, and protein timing must be prescribed in relation to the exertion effort ^16^. With the aim of optimizing protein synthesis in swimmers, an intake of 0.3 g/kg of high bioavailable protein must be consumed after finishing a competition, strength training, or interval training session ^14^. 

It should be noted, that amounts higher than 0.3 g/kg do not increase the protein synthesis rate ^33^. Additionally, athletes should be encouraged to ingest this amount of protein in 4-5 times during the day ^34^. These recommendations are based on a study that showed a higher protein muscle synthesis response when athletes consumed 4 intakes of 20 g of high-quality protein in 3-hour intervals after training, compared to 2 intakes of 40 g or 8 intakes of 10 g in similar conditions ^34^. However, it is important to note that these studies were not in aquatics sports. 

Regarding nutrient timing, especially carbohydrate intake, it must be noted that after 2-4 hours of training, a higher muscular glycogen resynthesis potential exists, thanks to a higher sensitivity to insulin action during this period, together with a higher activity of the glycogen synthase enzyme activation ^35^. The activation occurs because of the increment in the presence of calcium from the successive action potentials that take place during the effort ^36^. The higher glycogen synthesis rate takes place when carbohydrates intake reaches 1 g/kg/hour ^3^^,^^16^. When the goal between training sessions is to recover muscle glycogen stores, as well as favor protein synthesis, 1 g/kg of carbohydrate together with 20-25 g of protein should be ingested ^36^. On the contrary, when the goal of the training sessions is to achieve maximal efficacy of fat and carbohydrate metabolism, 0.3 g/kg of protein alone should be consumed. Lower amounts of carbohydrates (6-8 g/kg/day versus 10-12 g/kg/day) should also be consumed, once the period of glycogen synthesis is promoted, which will cause incomplete muscle glycogen stores. The state of incomplete muscle glycogen storage is optimal for lower intensity sessions ^37^, due to the fact that the transcription of genes that underlie the trainings gets favored, with the objective of improving fat and carbohydrate metabolism efficiency ^38^. 

Regarding fat intake, we must consider that these, besides their energetic function, play an important immunologic role ^39^. In this way, a diet with a low-fat content, can increase inflammatory cytokine levels, decrease antioxidant potential and negatively affect blood lipoprotein levels ^40^. The fat content in the diet is adjusted once the protein and carbohydrate content has been adjusted, although, the fat intake should represent at least a 20-25% of the energy intake ^13^, since, it would be difficult for diets with lower fat content to reach the essential fatty acids and liposoluble vitamin requirements ^41^. Furthermore, as it has been proved an association between the injury rates in women athletes and the lipid intake ^42^, it is possible that a deficient fat intake could cause sport injuries, although these data correspond in female runners and non-swimmers. 

A diet higher in fat content during periods of high volume and low-intensity training could maximize the contribution and efficiency of fatty acid metabolism during exercise ^31^^,^^43^. During low-intensity training session, it would be recommended to increase the intake of fats, up to 35% of daily energy intake. With respect to the type of fatty acids, saturated fatty acids should not exceed 10% of daily energy intake ^31^, while the intake of essential and monounsaturated fatty acids should be encouraged ^44^ (Table I). 

**Table 1:** Dietetic objectives of the different macronutrients according to the type of training performed in swimming.

Macronutrient	Type of session	Diary intake: 20-25% of energy intakeObjective
Carbohydrates	High volume and low intensity sessions	Diary Intake: 6g/kg/day
		Pre-training: Avoid carbohydrates intakes on the previous 2 hours
		Training: Avoid carbohydrates intakes
		Post-training: Intake of 1g/kg (if the next day there is a high intensity effort)
	High intensity sessions	Diary intake: 10-12g/kg/day
		Pre-training: 1-2g/kg on the previous 3-4 hours (avoiding ingesting on the 45 previous minutes)
		Training: 60-90g/hour (rate 2:1 between glucose:fructose) if the volume is high
		Post-training: Intake of 1g/kg (if the next day there is a high intensity effort)
Protein	Dairy recommendations	Diary Intake: 2g/kg/day
		Post-training: 0.3g/kg + 1g/kg of carbohydrates (if the next day there is a high intensity effort)
Fats*	High volume and low intensity sessions	Diary intake: 30-35% of energy intake
	High intensity sessions	Diary intake: 20-25% of energy intake

*The daily intake of saturated fatty acids should not overcome the 10% of the energy intake

### Hydration requirements

Although dehydration during water activities is lower than in other outdoor sports ^45^, due to thermoregulation improvement of different mechanisms from sweat as convection and conduction ^46^, the hydration needs of swimmers is higher than of the sedentary population ^47^. The higher the water temperature is, the higher the water loss in sweat will be ^46^. 

The first goal with regard to hydration is starting both training sessions and competitions in a euhydration state, which requires the athlete to hydrate in a rate of 5-7 ml/kg 4 hours prior to training, in addition to 3-5 ml/kg in case the athlete does not urinate during that time, or if the color of the urine is dark ^48^. The beverage should be cold, because cold beverage intake favors thermoregulation during swimming, especially when the temperature is high ^49^. 

When the losses of sweat are higher than 2% of body weight, and the amount of time in between training is less than 6-8 hours, it is necessary to establish a specific post training rehydration program ^3^^,^^16^. With the goal of optimizing hydration, and water retention (considering that some part will be excreted via diuresis), the amount of water should be equivalent to around 150% of fluid lost during training. Adding sodium to the beverage ^3^^,^^16^ could favor hydric retention, even if the co-ingested with carbohydrates. 

### Ergogenic Aids

In addition to adhering to an adequate nutrition plan, the intake of some nutritional supplements could favor both the athletes’ health status, as well as performance ^13^. Owing to the effect of supplement changes depending on the type of effort and sport modality ^50^, some supplements could have an ergogenic effect or show an improvement in performance for water sports. Swimmers are regularly reported to have high rates of supplement use. Even when there is an evidence base for the use of such products, the swimmer is advised to be aware of the appropriate situations and protocols of use, and to take into account the “cost” of products in terms of financial outlay and the small but real risk of an inadvertent doping outcome ^51^. The supplements and their effects are presented in Table 2. 

**Table 2:** Main ergogenic aids in swimming, effect and dosing.

Ergogenic Aid	Effect	Posology
Caffeine	Stimulator of the central nervous system, enhancer of the muscle contractility and reducer of the perceived exertion	≥3mg/kg 1 h before to the competition
Creatine	Improve the resynthesis of ATP through the high energy phsophagens system	3g/day during at least 4 weeks*
Sodium Bocarbonate	Acid-base balance regulator at a extracellular level	300 mg/kg 1–2 hour prerace
β-alanine	Acid-base balance regulator at a intracellular level, improving the activation of the myosin ATPase, sensibility to calcium on the muscle fibers and antioxidant function	6.4g/day in 4-8 takes of 0.8-1.6g in intervals of 1.5-3 hours
Nitrate	Precursor of the nitric oxide, improving the blood flow and the mitochondrial respiration	6-8mmol of nitrate (contained in betroot juice) in the 150-180 previous minutes to the competition and training*
Vitamin D	Bone remodelling, immune function and muscular contraction and new protein synthesis regulator	10μg/day*
Bovine Colostrum	Improvement of immune function	20g/day*
HMB	Anti-catabolic effect and precursor of the cholesterol and coenzyme A synthesis	3g/day during high intensity training sessions and/or during short periods of recovery in between training sessions*

*Recommendations adapted from other sports modalities

### Caffeine

Caffeine (1,3,7, trimethylxanthine) is an alkaloid from the family of the methylated xanthines, antagonist of the adenosine receptor ^52^. Caffeine intake in the athlete’s diet is common, as it is found in a wide variety of foods, such as coffee, tea, chocolate, energy drinks, and gels. Caffeine is quickly absorbed at the intestinal levels ^53^, detecting high concentration levels 15-45 minutes post intake, and maximum levels in the blood an hour post intake ^54^. 

Although studies, like the one published by Costill et al. show how supplementing with caffeine stimulates the rise of fatty acids in the blood, and thereby delaying period within which glycogen storage depletion occurs ^55^, there is no sufficient evidence to justify this hypothesis at the moment ^56^. 

Nevertheless, caffeine consumption can cause other side effects that could improve sport performance, as it stimulates the central nervous system due to the adenosine antagonistic effect ^53^, increases catecholamine levels ^57^, improves muscle contractility ^58^, improves action potential ^59^, as well as decreases the perceived exertion ^60^. Nowadays, caffeine is actually considered a supplement with ergogenic effects both in cardiorespiratory endurance, as well as in strength ^61^. 

Although there are few studies found on the consumption of caffeine in swimmers, there is evidence that prerace caffeine supplementation may enhance swimming performance ^62^^,^^63^, but further studies into swimming sports are required to know the dose and timing of prerace caffeine intake that are effective for swimming events, since recent studies suggest that the ergogenic benefit of taking caffeine alone for repeated 200-m swimming performance appears limited ^64^. 

There is some evidence from endurance sports protocols that small to moderate doses of caffeine (1–3 mg/kg) are as effective as larger doses (5–6 mg/kg) in achieving benefits and may reduce risk of side effects ^51^. Recently, Goods et al. had concluded that, a moderate dose of caffeine (3 mg/kg) ingested 1 h before a repeat sprint freestyle set significantly improves mean sprint time in elite swimmers, and the combination of at least a moderate dose of caffeine (>3 mg/kg) with trained athletes appears the most likely to result in ergogenic benefit to anaerobic exercise performance ^65^. 

Caffeine may be consumed in drinks or as an ingredient in some sport products (e.g., some gels). In this way, a recent study had concluded that a caffeinated energy drink increased some aspects of swimming performance in competitive sprinters, whereas the side effects derived from the intake of this beverage were marginal at this dosage (3 mg/kg) ^66^. 

Further research is required to determine the precise mechanism(s) responsible for caffeine’s ergogenic potential for anaerobic exercise performance. 

### Creatine

The purpose of supplementing with phosphocreatine is to increase the concentrations of muscular creatine, with the goal of maximizing the resynthesis of ATP through the phosphagen system ^8^. This process depends on the availability of muscular creatine ^67^. The metabolic pathway will have a great impact on performance, like high-intensity training carried out in most sports. During high-intensity training, creatine supplementation allows for higher training loads, which leads to higher training adaptations ^8^. 

Studies conducted specifically with the swimming population, showed that the supplementation of 20 g/day over 6 days, did not achieve any improvement in a group of trained swimmers, in a test of 6 x 50 m ^68^, or with a 25 g/kg supplementation during 4 days in a test of 10 x 50 m ^69^. A literature review therefore concluded that there was not enough evidence to recommend supplementing with creatine to improve performance in swimming tests, although it could improve performance during interval session in the swimming pool ^70^. The most likely benefits for swimmers come from using creatine in the training phase to enhance training adaptations to interval and resistance training. However, while there are studies that report an enhancement of the performance of such sessions in highly trained swimmers ^71^, there is some controversy about it at the moment ^72^. 

Nevertheless, because a higher quality in training means higher adaptation, creatine could be useful for water sports, especially during periods of high intensity training. The recommended dosage is 3 g/day, since it showed the same effects in 4 weeks with a load protocol of 5 days (20-30 g/day), followed by maintenance dosage of 3 g/day, compared to continued intake of 3 g/day ^73^, although this should be studied in swimmers. 

### Sodium bicarbonate

The increase in H+ derived from lactic acid dissociation, leads to a decrease in blood and muscle pH ^74^, disturbing the glycolytic rate ^75^, the uptake and reuptake of calcium in the sarcoplasmic reticulum ^76^, decreasing the contractile speed, while at the same time, causing perceived exertion to increase ^77^. These mechanisms aim to decrease the glycolytic contribution to metabolism, most likely as a defense mechanism facing a hypothetic extreme acidosis situation. Supplementing with sodium bicarbonate allows the release of H+ ions into the bloodstream, avoiding a situation of extreme acidosis in the intracellular compartment, which could inhibit muscular contractions ^78^, thus considering it as the main regulating agent of the acid-base balance at the extracellular level ^51^. It has been shown that supplementing swimmers with sodium bicarbonate, increases levels of sodium bicarbonate and pH in the blood, increases performance in 50-m swim tests ^79^, 200 m ^80^, successive sets of freestyle 100 m ^81^, and increases the average time in a 4-repetition set of freestyle 50-m swim ^79^. Since supplementing with sodium bicarbonate can increase performance of repeated sprints ^81^^,^^82^, it could also improve the quality of trainings, allowing the body to adapt ^3^. 

The acute use of bicarbonate to increase extracellular buffering capacity (300 mg/kg 1–2 hour prerace) might enhance the performance of 200–800 m swimming events via increased tolerance to the production of H+ ions via anaerobic glycolysis ^51^. Further field studies are needed with high-level swimmers since the few available studies show controversies ^64^^,^ ^83^^,^^84^. 

### β-alanine

The main purpose of supplementing with b-alanine is to increase muscular carnosine levels. The synthesis of carnosine depends on the bioavailability of the dietary intake of b-alanine ^85^. Carnosine, just like sodium bicarbonate, plays an important role with pH regulation, but acts differently than sodium bicarbonate, in that it acts at an intracellular level ^86^. In addition to its acid base regulation effect, carnosine has also shown to improve performance, through improving the Myosin ATPase activation ^87^, as well as improve calcium sensibility on muscular fibers ^88^ because of its antioxidant function ^89^. 

In swimmers, chronic supplementation with B-alanine to increase muscle carnosine, an intracellular buffer, may provide an alternative or additive strategy to support training adaptations and race performance ^51^. In a 4-week study performed on swimmers, the effect of 300 mg/kg of sodium bicarbonate, and 4.8 g/day of b-alanine in two tests of 100-m freestyle showed, that b-alanine alone did not affect performance, while the supplementation of b-alanine with sodium bicarbonate did improve performance on the second set of 100 m ^81^. It should be noted that even though b-alanine did not show an increase in performance, the recommended dosage is 6.4 g/day, for a period of 4-6 weeks ^90^, which is higher than what was used during the study. In relation to this, another study with highly trained swimmers who were supplemented with 6.4 g/day of b-alanine for 5 weeks, showed positive effects on performance in 100 m, and a tendency to improve in 200 m freestyle ^91^. 

It is, therefore, recommended to supplement 6.4 g/day of b-alanine for 4 or 6 weeks taken in 4-8 dosages of 0/8-1/6 g, with the aim of avoiding the main secondary effect from the supplement (paresthesia) ^90^. 

### Nitrate

The intake of 300-400 mg/day of nitrate, in the form of sodium nitrate ^92^^,^^93^, or beetroot juice (6-8 mmol of nitrate) ^94^, has demonstrated a doubling or tripling of nitrate plasma levels. The increase in nitrate plasma levels can lead to a simultaneous increase of nitric oxide levels, which regulates the muscle blood flow, as well as regulates the mitochondrial respiration ^82^. Supplementing with nitrate has shown to decrease VO2, for the same intensity effort ^92^. Supplementing with nitrate decreases the magnitude of the slow component of the VO2 ^95^^,^^96^, increasing the time until exhaustion at submaximal intensities ^97^. 

Although no studies on nitrates on aquatic spot modalities exist, it is feasible to assume that for these athletes who require a lower VO2 when employing a specific power output level, this supplement could have an ergogenic effect. This can especially be of importance in aquatic sports where a higher time in apnea (synchronized swimming) or swimming tests of longer distances in swimming pools or open waters is required. It is, therefore, recommended to supplement with nitrate salts, or beetroot juice (6-8 mmol of nitrate) in the 150-180 minutes prior to training sessions ^98^

### Vitamin D

Vitamin D has multiple functions related to sports performance, bone remodeling being one of them ^99^, improving muscle contractions, synthesizing new muscle proteins, as well as enhancing immune function ^100^. A study found that one third of a group of high performance swimmers had low serum levels of this vitamin, placing aquatic sports athletes at risk of developing hypovitaminosis ^100^. It may be caused due to limited sun light exposure, as most of the training takes place in indoor facilities, and high-level athletes usually dedicate the rest of the day to sedentary activities to aid recovery. This may explain the lower levels of serum vitamin D particularly found in swimmers, compared other sport modalities that train outdoors ^101^. 

Owing to the fact that optimal levels of vitamin D are linked with higher bone mineral density ^102^, it is assumed that this vitamin is important for optimal bone mineral density ^99^; therefore, lower levels of this vitamin could place the athlete at risk of injuries ^39^. To avoid the negative effects on performance that are associated with a deficit of this vitamin ^10^, swimmers should periodically check their serum levels in case supplementation is necessary if presented with suboptimal levels, and consume doses 10 μg/day approximately ^100^^-^^103^. 

### Bovine colostrum

Bovine colostrum is the milk produced by cows after they give birth to their calves, and it is known as a substance rich in immunoglobulins and growth factors ^104^. Because aquatic sports athletes frequently suffer from respiratory tract infections, it has been proposed that supplementing with bovine colostrum could improve the immune system, decreasing the risk of immunosuppression, and respiratory tract infections ^39^. 

In some studies, supplementation with bovine colostrum has demonstrated improvements in performance in cardiorespiratory endurance tests ^105^^,^^106^, as well as speed and strength ^107^. On the contrary, other research could not prove positive effects supplementing with bovine colostrum on strength or speed performances ^108^. 

Improvements in performance study were observed in a 12-minute cycloergometer test after ingesting 20 g/ day of bovine colostrum compared to 60 g/day ^106^, and thus, it is feasible to suggest supplementing with 20 g/day of bovine colostrum, with the goal being to improve the immune function, and possibly improving performance. 

### β-hydroxy-βmethylbutyrate

β-hydroxy-βmethylbutyrate (HMB), is a bioactive metabolite formed from the decomposition of leucine, an essential branched-chain amino acid ^109^. Although the body has the ability to synthesize HMB, the amount of leucine necessary to synthesize 3 g of HMB is 60 g of leucine, which would amount to 600 g of high biological value protein ^110^. The most important function of HMB for athletes is its anti-catabolic effect, improving protein synthesis, and inhibiting proteolysis ^111^. Research has demonstrated that supplementing with 3 g/day of HMB during high-intensity training sessions and/or during short periods of recovery in between training sessions, as could be in some aquatic sports modalities, can improve performance. The supplement has shown to improve health, performance, as well as increasing muscle strength ^111^^,^^112^^,^^113^. 

## Conclusions

High-level performance swimmers have a high energy expenditure, due to high training volume that they must try to balance with adequate energy intake. Dietary intake of carbohydrates should range between 6 g/kg/ day on days of low-intensity training, and 10-12 g/kg/ day on the days prior to high-intensity training sessions. Dietary protein intake should be at 2 g/kg/day for swimmers, while fat should be consumed at 20-25% of daily energy intake on days of high-intensity training, and 30- 35% on days of high volume and low-intensity training. In addition to having an adequate hydration plan in place before, during, and after training to help improve performance, and aid in recovery, the implementation of supplements at the right dosage, can enhance the athlete’s health, performance, and recovery. These include, caffeine, creatine, sodium bicarbonate, B-alanine, beetroot juice, vitamin D, bovine colostrum, and HMB. 

## Limitations

Although most of the nutritional guidelines recommended in this article have been extracted from swimmer articles, some of these recommendations are not from swimming studies and are from a limited case in swimming. These limitations result from an inadequate sample in sex, age, training status, and sports modalities. 
